# Astaxanthin Attenuates the Apoptosis of Retinal Ganglion Cells in *db/db* Mice by Inhibition of Oxidative Stress

**DOI:** 10.3390/md11030960

**Published:** 2013-03-21

**Authors:** Ling-Yan Dong, Jie Jin, Gao Lu, Xiao-Li Kang

**Affiliations:** 1 Department of Ophthalmology, Xinhua Hospital, School of Medicine, Shanghai Jiaotong University, Shanghai 200092, China; E-Mail: donglingyan_nj@yeah.net; 2 Department of Endocrinology, Xinhua Hospital, School of Medicine, Shanghai Jiaotong University, Shanghai 200092, China; E-Mail: jinjie_nj@yeah.net; 3 College of Life Sciences, Northeast Agricultural University, Harbin 150010, Heilongjiang, China; E-Mail: gaolu@neau.edu.cn

**Keywords:** retinal ganglion cells (RGCs), oxidative stress, apoptosis, astaxanthin

## Abstract

Diabetic retinopathy is a common diabetic eye disease caused by changes in retinal ganglion cells (RGCs). It is an ocular manifestation of systemic disease, which affects up to 80% of all patients who have had diabetes for 10 years or more. The genetically diabetic *db/db* mouse, as a model of type-2 diabetes, shows diabetic retinopathy induced by apoptosis of RGCs. Astaxanthin is a carotenoid with powerful antioxidant properties that exists naturally in various plants, algae and seafood. Here, astaxanthin was shown to reduce the apoptosis of RGCs and improve the levels of oxidative stress markers, including superoxide anion, malondialdehyde (MDA, a marker of lipid peroxidation), 8-hydroxy-2-deoxyguanosine (8-OHdG, indicator of oxidative DNA damage) and MnSOD (manganese superoxide dismutase) activity in the retinal tissue of *db/db* mouse. In addition, astaxanthin attenuated hydrogen peroxide(H_2_O_2_)-induced apoptosis in the transformed rat retinal ganglion cell line RGC-5. Therefore, astaxanthin may be developed as an antioxidant drug to treat diabetic retinopathy.

## 1. Introduction

Diabetic retinopathy is well-known as the result of microvascular retinal changes [1]. Hyperglycemia-induced intramural pericyte death and thickening of the basement membrane leads to incompetence of the vascular walls. These damages change the formation of the blood-retinal barrier and also make the retinal blood vessels become more permeable [2]. However, recent studies have shown that the retina has a high content of polyunsaturated fatty acids and has the highest oxygen uptake and glucose oxidation relative to any other tissue. This phenomenon renders retina more susceptible to oxidative stress [3]. And many diabetes-induced metabolic abnormalities were implicated in its development, and appeared to be influenced by elevated oxidative stress; however, the exact mechanism of its development remained elusive [4]. Oxidative stress plays an important role in diabetic complications. Retina and capillary cells experience increased oxidative damage in the diabetic milieu, and the antioxidant defense mechanism is impaired [5]. In hyperglycemia condition, the over-oxidation of lipid or glucose leads to increase the level of reactive oxygen species and induce oxidative stress in tissues. The possible sources of oxidative stress in diabetes might include auto-oxidation of glucose, shifts in redox balances, decreased tissue concentrations of low molecular weight antioxidants such as reduced glutathione (GSH) and vitamin E, and impaired activities of antioxidant defense enzymes such as MnSOD (manganese superoxide dismutase) and catalase [6]. In addition, oxidative stress inactivated VEGF survival signaling in retinal endothelial cells via PI 3-kinase tyrosine nitration [7]. Therefore, oxidative stress reflects an imbalance between the systemic manifestation of reactive oxygen species and a biological system’s ability to readily detoxify the reactive intermediates or to repair the resulting damage. Reactive oxygen species (ROS) generated by high glucose are considered as a causal link between elevated glucose and the other metabolic abnormalities important in the development of diabetic complications [8].

Apoptosis is a form of programmed cell death, which enabled a cell to direct its own destruction. This form of cell death appeared to be crucial for mammalian development and subsequent tissue homeostasis. In addition, oxidative stress induced caspase-independent apoptosis of retinal ganglion cells *in vitro* [9]. Ceramide is the key mediator of oxidative stress-induced apoptosis in retinal photoreceptor cells [10]. Apoptosis had been evidenced directly and the loss of functional cells was observed in diabetic patients’ retina. Thus, the induced apoptosis, such as in retinal ganglion cells or photoreceptor cells, played an important role in diabetic retinopathy.

Astaxanthin is a naturally occurring carotenoid with strong antioxidant properties both *in vitro* and *in vivo* [11]. It has been reported that astaxanthin restored the enzymatic antioxidant profile in salivary gland of alloxan-induced diabetic rats [12], and protected retinal cells against oxidative stress *in vitro* and in mice *in vivo* [13]. It was reported that in proximal tubular epithelial cells (PTECs), astaxanthin had a protective efficacy against several deleterious effects caused by high glucose exposure and proposed that astaxanthin should be explored further as a potential antidiabetic remedy for the treatment of diabetic nephropathy [14].

Based on the observations described above, the aim of the present study was to investigate the effects of astaxanthin on diabetic retinopathy in *db/db* mice with the reduction of oxidative stress. 

## 2. Results and Discussion

### 2.1. Results

#### 2.1.1. Astaxanthin Improves Oscillatory Potentials(OPs) in *db/db* Mice

**Figure 1:**
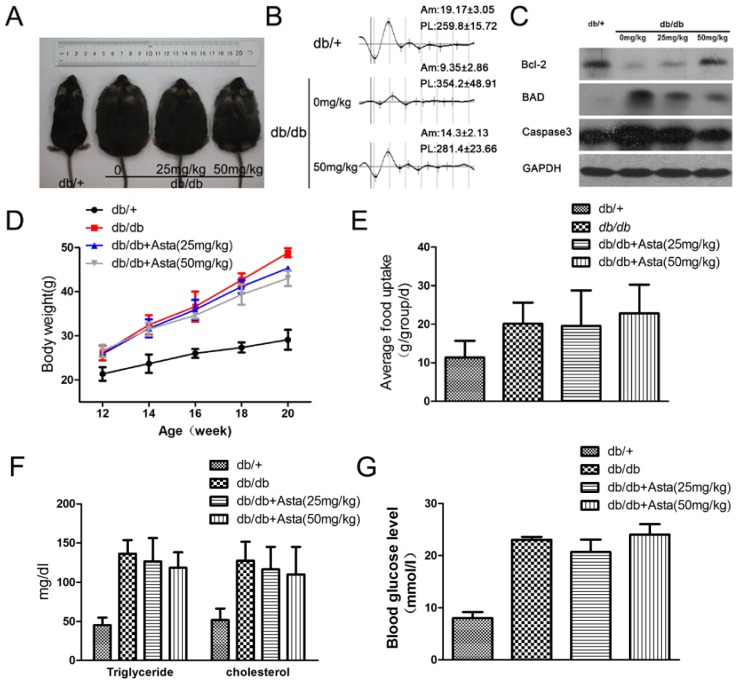
The effect of astaxanthin on retinal function in *db/db* mice. Male mice were injected (i.g.) with astaxanthin for eight weeks (week 12–20). Representative images of male mice are shown for all groups (*n* = 8 per group) (**A**). Representative ERG traces from three groups; Am, amplitude (μV); PL, peak latency (mS) (**B**). Retinal electroretinogram (ERG) was recorded from 8 mice per group, at 12–14 weeks of age in response to a series of light flashes at increasing intensity. Treatment with astaxanthin for 8 weeks reduced the expression of apoptosis gene proteins in the retina as determined by Western blot (**C**). Weight gain and food intake in all groups (**D**,**E**). Quantitative analysis of plasma triglyceride and cholesterol (**F**). The random-blood glucose test (**G**) were performed 2 h after treatment with astaxanthin.

It is known that carotenoids in plants have potential anti-oxidant effects and have also been used to prevent a variety of diseases in diets [15,16,17]. We investigated the effect of astaxanthin on *db/db* mice. The mice treated with 25 or 50 mg/kg astaxanthin per day for eight weeks showed loss less of body weight gain, and the amount of food intake was not significantly changed during drug therapy (Figure 1A,D,E). For the frequency spectrum and amplitude analysis of dark- and light-adapted, oscillatory potentials (OPs) of *db/db* mice was reduced in amplitude (Am) and peak latency (PL), and astaxanthin reversed such effects (Figure 1B). Astaxanthin treatment resulted in a decline of blood fat level but the differences were not statistically significant among the experimental groups (Figure 1F). To evaluate the effect of astaxanthin on glucose metabolism, random-blood glucose test were conducted after treatment with astaxanthin for 2 h. As shown in Figure 1G, astaxanthin could not improve the impaired glucose tolerance and increase glucose uptake *in vivo*. These results suggested that astaxanthin could potentially improve retinal function in the development of diabetes in *db/db* mice.

#### 2.1.2. Astaxanthin Inhibits the Apoptosis of Retinal Ganglion Cells (RGCs) in *db/db* Mice

**Figure 2:**
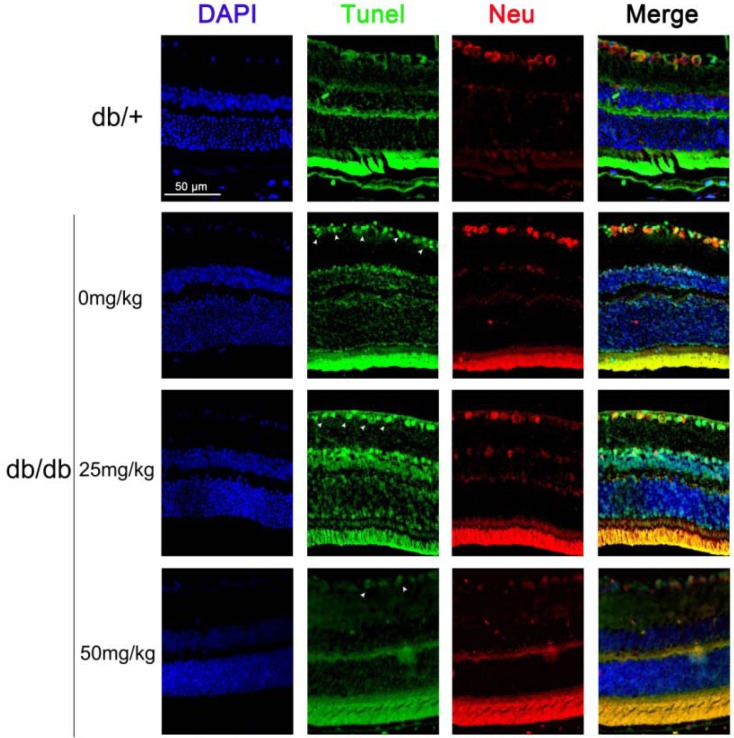
The effect of astaxanthin on the apoptosis of RGCs in *db/db* mice. After treatment for eight weeks, animals were sacrificed. The retina samples were prepared for staining assay. Apoptotic cells were marked with green fluorescence, the nuclei of cells are stained by blue fluorescence (DAPI) and RGCs are stained by red fluorescence (Neu, neuron).

Maintenance of functional cell mass is critical for signal transduction. Dysfunction induced by the decreased population of cells is regarded as an important factor in the pathogenesis of various metabolic diseases [18,19]. To determine the effects of astaxanthin on RGCs, the apoptosis was determined using tunel assays. The detection of DNA damage by tunel staining indicated that astaxanthin decreased the apoptosis of RGCs in retina (Figure 2). As shown in Figure 1C, we investigated the protein levels of apoptosis genes by Western blot and the expression level of Bcl-2, BAD and caspase-3 proteins had significantly changed in retina of 20-week *db/db* mice, compared with *db/+* mice, and astaxanthin reversed such effects. These data indicated that the treatment with astaxanthin could reduce the apoptosis of RGCs in *db/db* mice.

#### 2.1.3. Astaxanthin Reduces Oxidative Stress of the Retina in *db/db* Mice

**Figure 3:**
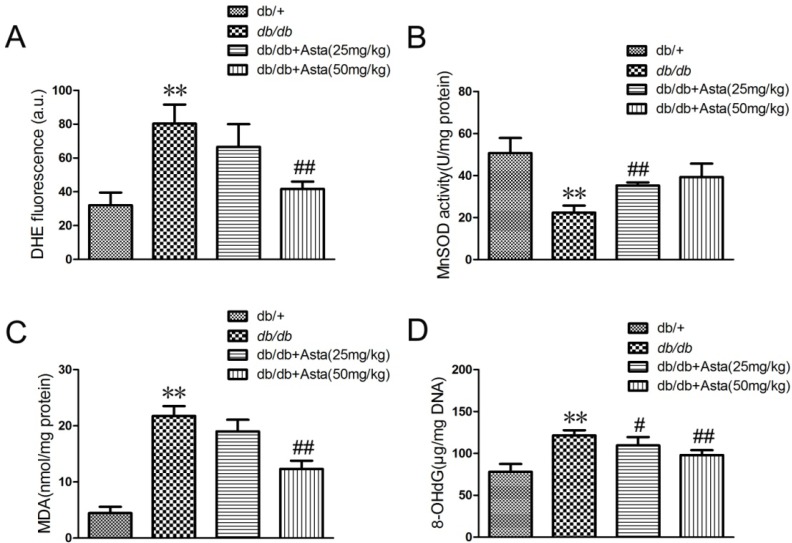
Effects of superoxide (DHE), MDA and 8-OHdG levels and MnSOD activity in the retinal tissue. After treatment for eight weeks, animals were sacrificed. The retina samples were prepared for quantitative analysis. (**A**) superoxide level (DHE), (**B**) MnSOD activity, (**C**) MDA production and (**D**) 8-OHdG level were measured according to the manufacturer’s instructions for each assay. Values are the means ± SD (*n* = 3) of three independent experiments.* *P* < 0.05, ** *P* < 0.01 *vs.* control (*db/+*), ^#^* P* < 0.05, ^##^* P* < 0.01 *vs. db/db*.

Oxidative stress has been implicated in the pathogenesis of diabetic retinopathy [20,21]. To estimate the anti-oxidative effect of astaxanthin, superoxide anion (DHE), MDA and 8-OHdG levels and MnSOD activity in the retina tissues were measured. As an initial indicator of ROS, we used DHE staining, which is a probe for O_2_^•−^ and produces red fluorescence as a result of the complex between ethidium and DNA as described by previous study [22]. To quantitate changes in O_2_^•−^ levels, we measured total DHE fluorescence in retinal homogenates. O_2_^•−^ levels was promoted in retina in *db/db* mice control, and astaxanthin reversed such effects (Figure 3A). MnSOD is a pivotal enzyme scavenging ROS *in vivo* and MnSOD activity in the retinal tissue was significantly increased after 8-week treatment (Figure 3B). Oxidative stress, as determined by the concentrations of MDA (as a marker of lipid peroxidation) and 8-OHdG (indicator of oxidative DNA damage), remained elevated in *db/db* mice control. Administration of astaxanthin inhibited the increase of MDA and 8-OHdG levels in retinal (Figure 3C,D). Therefore, astaxanthin showed an anti-oxidative effect in the retina of *db/db* mice.

#### 2.1.4. Astaxanthin Attenuates H2O2-Induced Apoptosis in the Transformed Rat Retinal Ganglion Cell Line RGC-5

**Figure 4:**
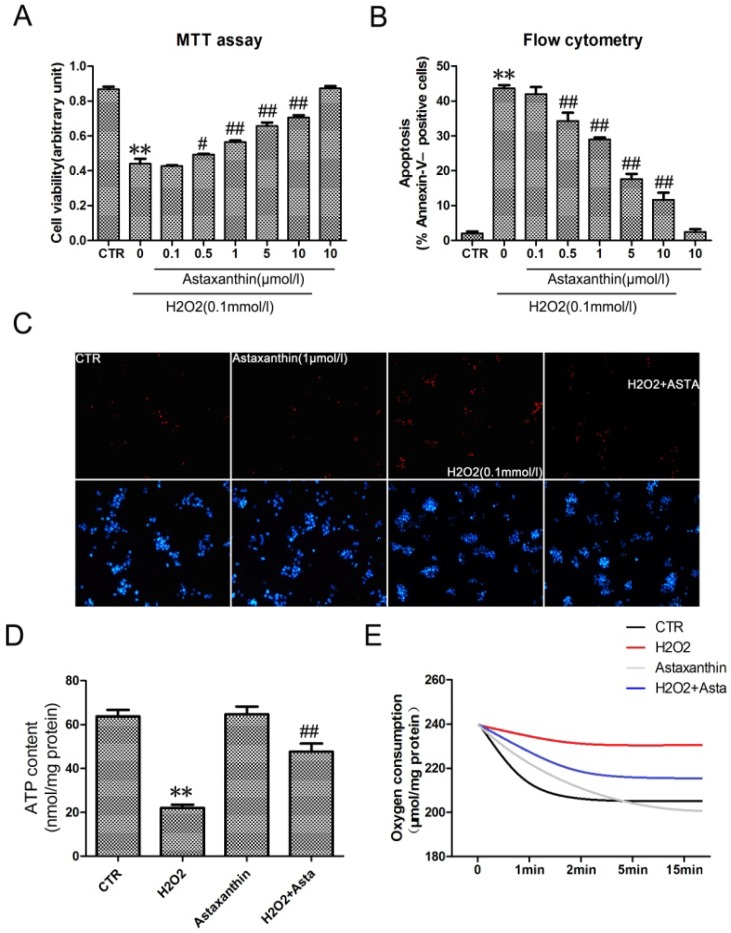
The effect of astaxanthin on H_2_O_2_-induced apoptosis in the transformed rat retinal ganglion cell line RGC-5. (**A**) Cells were treated with the indicated concentrations of astaxanthin and H_2_O_2_ (0.1 μmol/L) for 24 h, analyzed by MTT assay. (**B**) Cells werestained with Annexin V-FITC and PI, analyzed by flow cytometry. Data are expressed as % of Annexin V-FITC- positive and PI-negative cells (early stage of apoptosis). (**C**) Representative photographs of double staining of PI and Hoechst 33342. The apoptotic cells were observed as PI intense signal after double staining. (**D**) The intracellular ATP concentrations were evaluated after exposure to astaxanthin and H_2_O_2_ for 24 h. Before the oxygen consumption test (**E**), RGC-5 cells were pre-treated in low oxygen conditions for 2 h in suspension culture with astaxanthin and H_2_O_2_. Values are the means ± SD (*n* = 3) of three independent experiments. * *P* < 0.05, ** P < 0.01 *vs.* control, # *P* < 0.05, ## *P* < 0.01 *vs.* group treated with H_2_O_2_ (0.1 μmol/L).

More and more research pinpoints oxidative stress as a root cause of diabetic retinopathy [23,24]. To investigate the effect of astaxanthin on the apoptosis of RGC-5 cells, cell viability was determined in cells using MTT assays. Astaxanthin significant inhibited the decrease of RGC-5 cell viability induced by H_2_O_2_ in a dose-dependent manner (Figure 4A). Determination of DNA content by Hoechst/PI staining indicated that astaxanthin decreased the amount of dead cells in RGC-5 cells (Figure 4C). Quantitative evaluation of apoptosis through annexin V-FITC/PI staining was analyzed by Flow Cytometry. As shown in Figure 4B, the rate of apoptotic cells were risen to 43.47% with the treatment of H_2_O_2_ (0.1 mmol/L) for 24 h. Furthermore, pretreatment with astaxanthin prevented H_2_O_2_-induced apoptosis in a dose-dependent manner. As astaxanthin accelerated the amount of live cells, the production of ATP (Figure 4D) and the total oxygen uptake (Figure 4E) were also promoted significantly in RGC-5 cells, compared with the group treated with H_2_O_2_ (0.1 mmol/L). Taken together, it suggested that astaxanthin had a strong anti-apoptotic effect by inhibition of H_2_O_2_-induced oxidative stress in RGC-5 cells.

### 2.2. Discussion

As nature’s most powerful antioxidant, astaxanthin has been documented to show a wide range of benefits in human clinical studies on several serious health concerns [25]. Due to its molecular structure, astaxanthin is one of the few antioxidants that can move throughout the entire body and provide protection to all of our cells. In addition, astaxanthin has the unique property that it can protect the entire cell. It has polar hydrophilic ends that span across the entire cell membrane [26]. Most other well-known antioxidants cannot do this. Some results showed that astaxanthin protected neuronal cells against oxidative damage and is a potent candidate for brain food [27], and protected β-cells against glucose toxicity in diabetic *db/db* mice [28]. In addition, administration of astaxanthin might be a novel approach for the prevention of diabetes nephropathy [29]. However, the role of astaxanthin on diabetic eye disease is not clear. Our results provided a new insight into potential beneficial effects of antioxidants in the treatment of retinopathy in diabetic patients. Astaxanthin improved oscillatory potentials (OPs) and reduced oxidative stress of the retina in *db/db* mice. Moreover, astaxanthin attenuated H_2_O_2_-induced apoptosis in the transformed rat retinal ganglion cell line RGC-5. Therefore, astaxanthin may be used in diabetic retinopathy. 

It is well-known that the development of diabetes mellitus is caused by chronic multi-factors such as Gluco-lipotoxicity, inflammatory mediators and oxidative stress [30,31]. Many diabetes-induced metabolic abnormalities are implicated in its development, and appear to be influenced by elevated oxidative stress; the exact mechanism of its development remains elusive. In our studies, astaxanthin could decrease the level of oxidative stress that caused dysfunction, apoptosis and ATP depletion. However, astaxanthin could not improve the impaired glucose tolerance and increase glucose uptake in *db/db* mice (Figure 1G). It suggested that oxidative stress was one of important factors resulted in hyperglycemia. In addition, we think astaxanthin, as an antioxidant, shows its radical scavenging ability by decomposition of H_2_O_2_. So the effect of astaxanthin on the singaling of H_2_O_2_-induced apoptosis was not shown. However, astaxanthin significantly improved oscillatory potentials (OPs) *in vivo* in Figure 1. The oscillatory potentials (OPs) in electroretinogram (ERG) have clinical values in measuring retinal functions of the early stage of diabetic retinopathy [32]. The National Eye Institute data is very alarming; it suggests that about half of the people with diabetes in the United States have at least some form of retinopathy, and about 700,000 have some serious retinal disease. Diabetic retinopathy is affecting approximately 65,000 people in the United States alone causing 12,000 to 24,000 new cases of blindness each year [3]. Moreover, oxidative stress played the key role on diabetic retinopathy, which directly caused cell dysfunction or apoptosis in retinal ganglion cells (RGCs) [33,34] and activated VEGF survival signaling in retinal endothelial cells via PI 3-kinase tyrosine nitration [35]. Thus, oxidative stress and antioxidant defenses may be as a target for the treatment of Diabetic Eye Disease.

As shown in Figure 3, astaxanthin reversed the change of oxidative stress markers in the retinal tissue, including superoxide anion (DHE), MDA and 8-OHdG and MnSOD. Thus, it suggested that astaxanthin, as an antioxidant, possibly improved diabetic retinopathy by inhibition of oxidative stress.

The *db/db* mouse is a model of obesity, diabetes, and dyslipidemia wherein leptin receptor activity is deficient because the mice are homozygous for a point mutation in the gene for the leptin receptor [36]. In addition, oxidative stress has been implicated in the pathogenesis of diabetic complication in *db/db* mouse, such as retinopathy and nephropathy [24,37]. Some reports have evaluated visual response properties of retinal ganglion cells (RGCs) during the early stage of diabetic insult (8, 12, and 20 weeks) in *db/db* mice and determined that increased oxidative stress played a role in impaired visual functions of RGCs in 10–20 weeks old *db/db* mice [38]. As shown in Figure 2, apoptotic RGCs were significantly increasing in 20-weeks old *db/db* mice. Therefore, *db/db* mouse have developed as a model of early dysfunction and apoptosis of retinal ganglion cell.

## 3. Material and Methods

### 3.1. Reagents

Astaxanthin (HLPC content 98%) was purchased from Shanghai Winherb Medical S & T Development Co. Ltd. (Shanghai, China). Dulbecco’s modified Eagle media (DMEM) and fetal bovine serum (FBS) were purchased from Gibco (Carlsbad, CA, USA). ATP assay kit was obtained from Beyotime (Jiangsu, China). Terminal Transferase dUTP Nick End Labeling (TUNEL) Assay kit was purchased from Roche, USA. The Annexin-V-FITC Apoptosis Detection Kit was purchased from BD Biosciences, USA. Double Stain Apoptosis Detection Kit (Hoechst 33342/PI) was purchased from GenScript, USA. The rabbit polyclonal antibodies against Bcl-2, BAD and caspase-3 were purchased from Cell signal technology, USA. Dihydroethidium (DHE) was purchased from Molecular Probes, USA. Competitive ELISA kit for MDA, 8-OHdG and MnSOD was purchased from Cayman Chemical.

### 3.2. Animal Treatments

Male *db/db* mice (12 weeks old; C57BL/KsJ; *n* = 8 per group) were purchased from the Shanghai Institute of Materia Medica, Chinese Academy of Sciences. The animals fed with a normal diet were dosed daily by oral gavage with vehicle (0.5% carboxymethylcellulose) or selected test compounds suspended in 0.5% carboxymethylcellulose, at 25 or 50 mg/kg of Astaxanthin. After treatment for the indicated number of days, animals were sacrificed. The collected blood was centrifuged (3000 rpm, 5 min, 4 °C) to obtain plasma. All tissues were rapidly removed, placed in liquid nitrogen and stored at −80 °C for subsequent preparation of pathological sections. Animal studies were carried out according to the Guidelines for Animal Experiments, as outlined by the Committee for Animal Experiments of National Center.

### 3.3. Electroretinogram Recordings

Electroretinogram recordings were performed as previously established in this laboratory [39]. In brief, animals were dark adapted for two hours before being anesthetized with ketamine (62.5 mg/kg i.p.) and xylazine (12.5 mg/kg i.p.) under dim red lighting. Mouse body temperature was maintained at 38 °C using an electric heating blanket. Pupils were dilated using 1% tropicamide and then one drop of methylcellulose was added to prevent corneal dehydration and allow electric contact between the gold recording loop and the eye. Reference electrodes were platinum needles (25 G, Grass) implanted subcutaneously 2–3 mm posterior to the eye, while the ground electrode was a platinum needle inserted subcutaneously in the nape of the neck. Stimuli were white flashes of light (xenon, <2 ms) presented in a ColorDome full field ganzfeld (Diagnosys LLC, Lowell, MA, USA). Responses were recorded at 5000 Hz sampling frequency and amplified from 1 to 700 Hz with no notch filtering using the Espion E2 system (Diagnosys). Recordings lasted for 250 ms after the stimulus was presented, with a baseline of 40 ms before the flash. The scotopic intensity response was recorded in response to flashes ranging from −5.2 to 2.8 log cd s/m^2^, with each flash repeated 3–5 times and the responses averaged together. To allow for rod recovery, inter-stimulus interval ranged from 4 s at the dimmest flash to 60 s at the brightest flash.

### 3.4. Electroretinogram Analysis

The b wave amplitude was measured from the a-wave negative peak to the positive peak of the b-wave. If the peak of the b-wave coincided with the oscillatory potentials, the point halfway between the trough and peak of the highest OP was used as the b-wave amplitude. Oscillatory potentials were isolated from the averaged recording traces using a 75–300 Hz digital filter in the Espion E2 system. The peaks were numbered following Rousseau and LaChapelle [40] for OPs 2 through 5. Amplitude of the oscillatory potentials was measured from bottom of the trough preceding each OP peak, to the top of that peak. Criterion amplitudes were set as follows, to separate signal from noise: OPs and a-wave: 10 μV, b-wave: 20 μV. The response threshold was defined as the minimum stimulus intensity that elicits a response greater than the criterion amplitude. The frequency spectra and dominant frequency of isolated OPs were analyzed by performing a fast Fourier transform (ScopeDSP, Iowegian International) on traces recorded in response to a 2.4 log cd s/m^2^ flash on scotopic background as described in Yu and Peachey [41]. 

### 3.5. Blood Glucose Test

Random tests were performed at every week after the onset of Astaxanthin dosing. Mice were not fasted before test. Approximately 5 μL of whole blood was drawn from the tip of the tail vein, and glucose was measured with the Optium Xceed™ Diabetes.

### 3.6. Blood Lipid Assay

About 50 μL tail vein blood from overnight-fasted or ad libitum-fed mice was collected in EDTA-coated tubes. Plasma TG, FFA, and cholesterol levels were determined using a Roche Cobas blood chemistry analyzer.

### 3.7. Western Blot

Tissues sample harvesting as described above, and the lysed with ice-cold lysis buffer containing 50 mmol/L Tris-HCl, pH 7.4; 1% NP-40; 150 mmol/L NaCl; 1 mmol/L EDTA; 1 mmol/L phenylmethylsulphonyl fluoride; and complete proteinase inhibitor mixture (one tablet per 10 mL; Roche Molecular Biochemicals, Indianapolis, IN, USA). After protein content determination using a DC Protein Assay kit (Bio-Rad Laboratories, Hercules, CA, USA), western blot was performed as described previously [42].

### 3.8. Tunel Staining

Eyes were enucleated and placed in phosphate buffered 4% paraformaldehyde (FD Neurotechnologies, Inc., Baltimore, MD, USA) for 2–2.5 h. The eye was cut at the ora serrata and the anterior portion of the eye and lens were removed. The eyecup (retina, choroid, and sclera) was placed in 15%–20% sucrose solution at 4 °C overnight for cryoprotection. Eyecups were then placed in optimal cutting temperature (O.C.T.) compound and cut at thicknesses of 8 μm and stored at −80 °C until labeled. The apoptotic cells in tissues were stained by tunel staining kit following its protocol, the apoptotic cells were stained by green fluorescence, and all cells were marked with blue fluorescence using DAPI. The apoptotic ratio was calculated as tunnel-positive cells divided by total cell number.

### 3.9. Detection of the Level of Superoxide Anion (O_2_^•−^), MDA, 8-OHdG and MnSOD

We used the probe dihydroethidium (DHE; Molecular Probes, Eugene, OR, USA) to detect intracellular O_2_^•−^. O_2_^•−^ oxidizes DHE to ethidium, which generates a red fluorescent signal. Fluorescence spectrometry of tissue O_2_^•−^ production was performed according to previous methods [43].

The retina samples were prepared as a 10% homogenate in 0.9% saline using a homogenizer on ice according to their respective weight. Then the homogenate was centrifuged, and the supernatant was collected and diluted. The assay of the MDA levels was performed according to the manufacturer’s instructions for the MDA detection kit.

Competitive ELISA for 8-OHdG was performed according to the manufacture’s protocol. Sample DNA assays were performed in triplicate. Standard 8-OHdG was assayed over a concentration range of 0.125–20 ng/mL in duplicates for each experiment. The average concentration of 8-OHdG per microgram of DNA for each group was calculated for each sample. Controls without added DNA and appropriate blanks were also incorporated into experiments.

The enzyme activity of MnSOD was measured in 5–10 μg of retinal protein using a kit. The method utilizes tetrazolium salt to quantifying O_2_^•−^ generated by xanthine oxidase and hypoxanthine. The standard curve was generated using a quality-controlled SOD standard. MnSOD activity was determined by performing the assay in the presence of potassium cyanide to inhibit Cu–ZnSOD, thus measuring the residual MnSOD activity.

### 3.10. Cell Culture

RGC-5 cells (ATCC, VA, USA) were routinely maintained in DMEM supplemented with 10% fetal bovine serum (FBS; Gibco), 100 U/mL penicillin and 100 g/mL streptomycin (Gibco). Cells were grown in a humidified incubator of 95% air and 5% CO_2_ at 37 °C. Cells were passaged when 80% confluent.

### 3.11. MTT Assay

Cell viability was determined using 3-(4,5-dimethylthiazol-2-yl)-2,5-diphenyltetrazolium bromide (MTT) assay. Briefly, the cells were seeded in 96-well dishes at 1 × 10^4^ to 2 × 10^4^ cells per well, and pretreated with or without astaxanthin for 2 h. Each well was then supplemented with 10 μL MTT (Sigma) and incubated for 4 h at 37 °C. The medium was then removed, and 150 μL dimethyl sulfoxide (Sigma) were added to solubilize the MTT formazan. The optical density was read at 490 nm.

### 3.12. Flow Cytometry

To estimate the number of apoptotic cells, cells were fluorescently labeled by addition of 20 μL of binding buffer, 5 μL of Annexin V-FITC and 5 μL of propidium iodide. After the incubation at room temperature in dark for 15 min, cells were applied to flow cytometry analysis. A minimum of 10,000 cells in the gated region was analyzed by BD FACS Calibur Flow Cytometer. Results were interpreted by the percentage of total cells appearing in each quadrant.

### 3.13. Hoechst/PI Staining

Apoptotic cell death was evaluated by staining the non-viable cells red with propidium iodide (PI) and Hoechst 33342 (Sigma), which stained the nuclei of both live and dead cells blue. Staining with Hoechst allows for the discrimination of apoptotic cells based on nuclear morphology and evaluation of membrane integrity. The Hoechst dye was added to the culture medium at a ﬁnal concentration of 5 µg/mL, and the cultured cells were incubated at 37 °C for 30 min. The PI (5 μg/mL) solution was then also added immediately before observation in the ﬂuorescence microscope.

### 3.14. ATP Assay

The level of intracellular ATP was determined using the ATP Bioluminescence Assay Kit. Cultured cells were lysed with a lysis buffer, followed by centrifugation at 12,000× *g* for 1 min at 4 °C. Finally, the level of ATP was determined by mixing 50 μL of the supernatant with 50 μL of luciferase reagent, which catalyzed the light production from ATP and substrate. The emitted light was linearly related to the ATP concentration and measured using a microplate luminometer.

### 3.15. Oxygen Consumption

To evaluate the ability of cellular oxygen consumption (VO2) during drug treatment, the Micro Respirometry System (Strathkelvin, Mitocell S200) was used to measure oxygen content of culture media. Before the assay, primary hepatocytes just isolated from mice were pre-treated in low oxygen conditions for 2 h in suspension culture with drugs.

### 3.16. Statistical Analysis

Differences between groups were analyzed using the two-sided t test and ANOVA with *P* < 0.05 considered statistically significant.

## 4. Conclusions

In this study, we investigate the effects of astaxanthin on diabetic retinopathy in *db/db* mice with the reduction of apoptosis of retinal ganglion cells by inhibition of oxidative stress. Therefore, astaxanthin may be developed as a new drug to treat Diabetic Eye Disease from oxidative stress.
